# Supplementary figures and images for: Neuropeptide‐Dependent Spike Time Precision and Plasticity in Circadian Output Neurons
Source: Eur J Neurosci. 2025 Mar 13;61(5):e70037. doi: 10.1111/ejn.70037 (PMC11906214; doi:10.1111/ejn.70037)

(a)

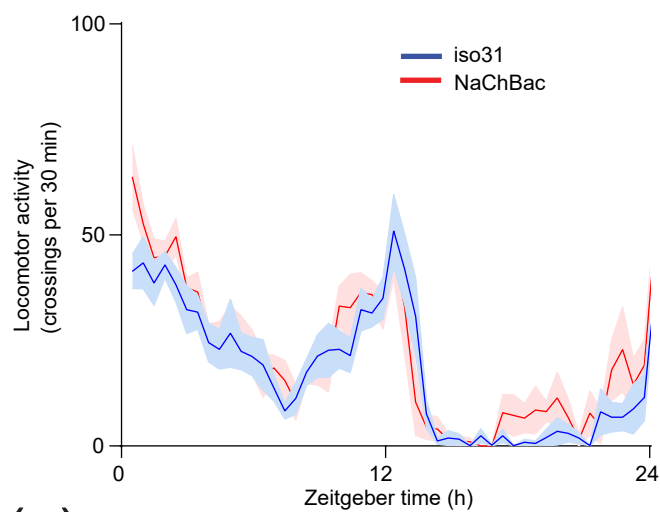

(b)

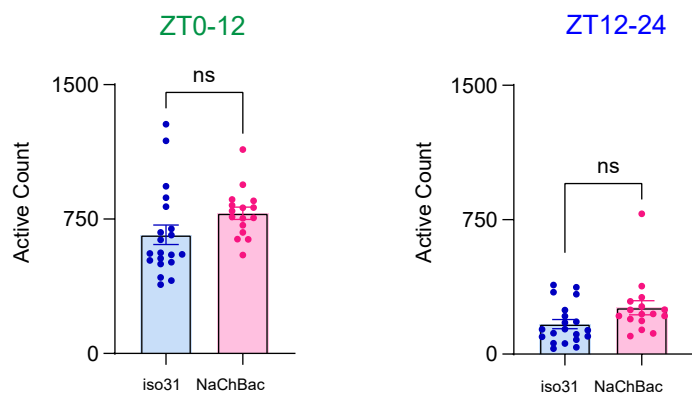

(c)

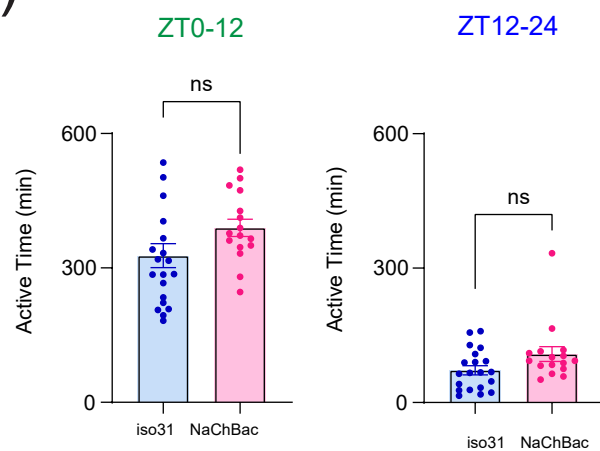

(d)

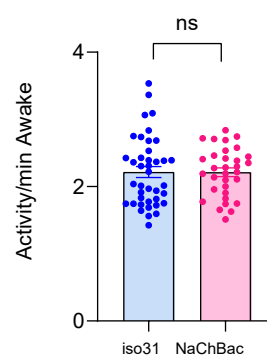

(e)

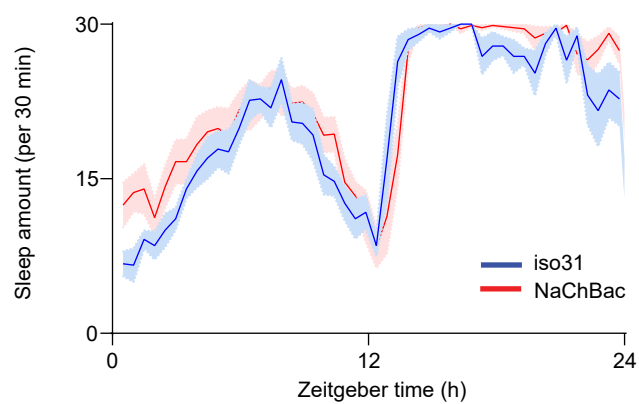

(f)

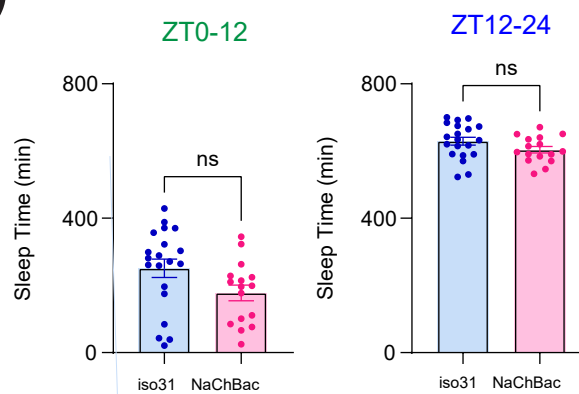

Supplementary Figure S1

Supplement: Supplementary file 1 — Figure S1 (a) Activity profiles. (b) Activity count at ZT0–12 and ZT12–24. (c) Active time at ZT0–12 and ZT12–24. (d) Daily waking activity of iso31 (blue) and UAS‐NaChBac alone control (red) flies. (e) Sleep profiles. (f) Sleep time at ZT0–12 and ZT12–24 of iso31 (blue) and UAS‐NaChBac alone control (red) flies. Sleep time plotted in 30 min bins. The statistics used were unpaired t‐tests, and ns indicated non‐significant. [file EJN-61-0-s001.pdf]

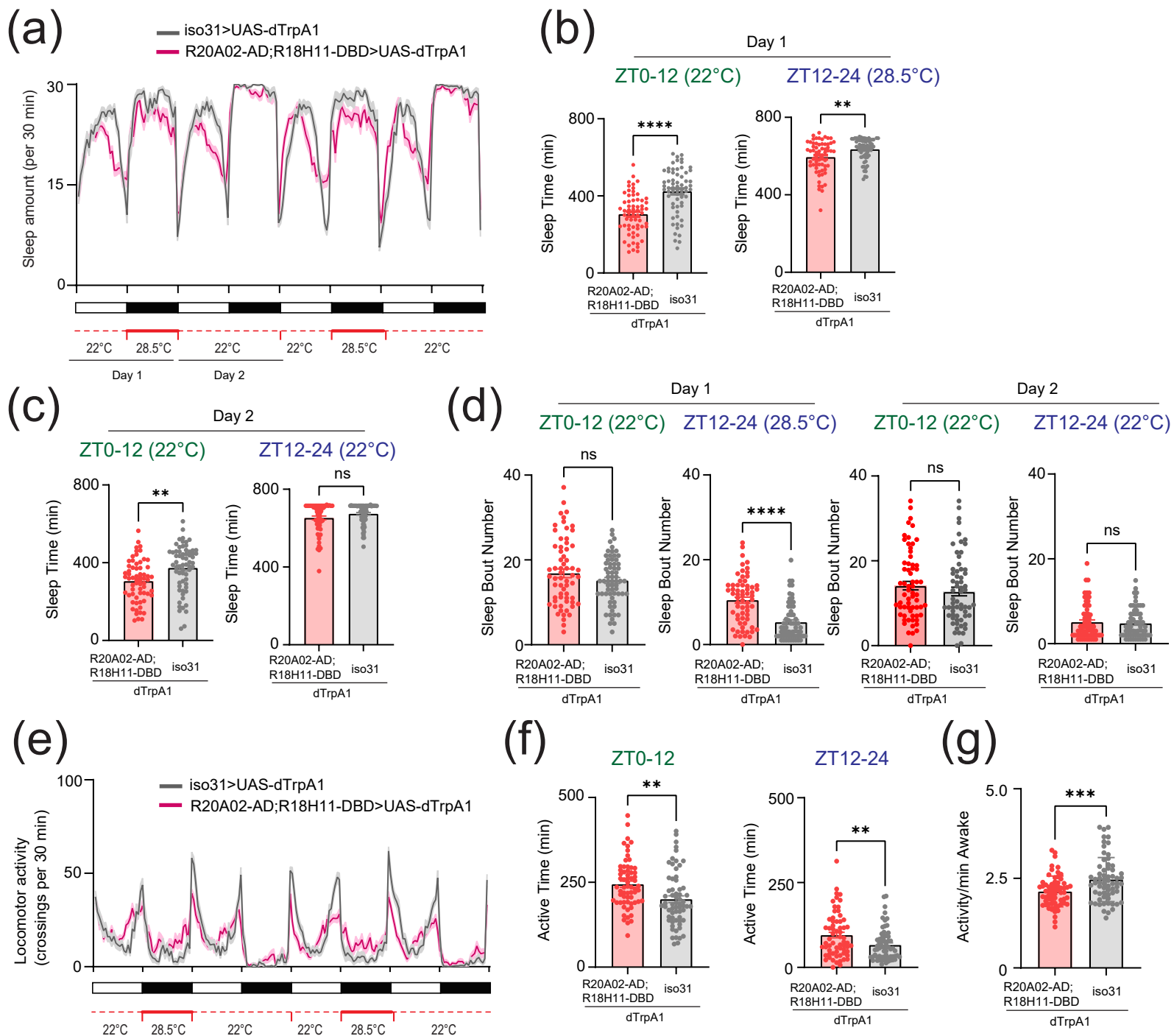

Supplementary Figure S2

Supplement: Supplementary file 2 — Figure S2 (a) Sleep profiles. (b) Sleep time at ZT0–12 and ZT12–24 on Day 1 having temperature elevation. (c) Sleep time at ZT0–12 and ZT12–24 on Day 2 without temperature elevation. (d) Sleep bout number of UAS‐dTRPA1>iso31 control (gray) and R20A02‐ad;R18H11‐DBD>UAS‐dTRPA1 (red) flies. (e) Activity profiles. (f) Active time at ZT0–12 and ZT12–24. (g) Daily waking activity of UAS‐dTRPA1>iso31 control (gray) and R20A02‐ad;R18H11‐DBD>UAS‐dTRPA1 (red) flies. The statistics used were unpaired t‐test with **p < 0.01, ***p < 0.001, and ****p < 0.0001, and ns indicated non‐significant. [file EJN-61-0-s002.pdf]
