# Supplementary material for: Neuropeptide‐Dependent Spike Time Precision and Plasticity in Circadian Output Neurons
Source: Eur J Neurosci. 2025 Mar 13;61(5):e70037. doi: 10.1111/ejn.70037 (PMC11906214; doi:10.1111/ejn.70037)
Supplement: Supplementary file 3 — Table S1 Details for statistical tests in Figures S1 and S2. [file EJN-61-0-s003.docx]

**Table S1. Details for statistical tests in Supplemental Figures S1 and S2**

| **Figure** | **Comparisons** | **Statistical Test** | ***P*** | **Test Statistics** | **Replicates** |
| --- | --- | --- | --- | --- | --- |
| S1b | Active count at ZT0-12 of flies with *iso31* or *UAS-NaChBac* | Unpaired t-test | 0.0869 |  | iso31: 20  NaChBac: 16 |
|  | Active count at ZT12-24 of flies with *iso31* or *UAS-NaChBac* | Unpaired t-test | 0.0513 |  | iso31: 20  NaChBac: 16 |
| S1c | Active time at ZT0-12 of flies with *iso31* or *UAS-NaChBac* | Unpaired t-test | 0.0806 |  | iso31: 20  NaChBac: 16 |
|  | Active time at ZT12-24 of flies with *iso31* or *UAS-NaChBac* | Unpaired t-test | 0.0582 |  | iso31: 20  NaChBac: 16 |
| S1d | Activity/min awake of flies with *iso31* or *UAS-NaChBac* | Unpaired t-test | 0.9745 |  | iso31: 40  NaChBac: 32 |
| S1f | Sleep time at ZT0-12 of flies with *iso31* or *UAS-NaChBac* | Unpaired t-test | 0.0544 |  | iso31: 20  NaChBac: 16 |
|  | Sleep time at ZT12-24 of flies with *iso31* or *UAS-NaChBac* | Unpaired t-test | 0.1152 |  | iso31: 20  NaChBac: 16 |
| S2b | Sleep time at ZT0-12 of flies with *R20A02-AD;R18H11-DBD dTrpA1* or *iso31 dTrpA1* (Day1) | Unpaired t-test | <0.0001 |  | 64 per genotype |
|  | Sleep time at ZT12-24 of flies with *R20A02-AD;R18H11-DBD dTrpA1* or *iso31 dTrpA1* (Day1) | Unpaired t-test | 0.0016 |  | 64 per genotype |
| S2c | Sleep time at ZT0-12 of flies with *R20A02-AD;R18H11-DBD dTrpA1* or *iso31 dTrpA1* (Day2) | Unpaired t-test | 0.01 |  | 64 per genotype |
|  | Sleep time at ZT12-24 of flies with *R20A02-AD;R18H11-DBD dTrpA1* or *iso31 dTrpA1* (Day2) | Unpaired t-test | 0.07 |  | 64 per genotype |
| S2d | Sleep bout number at ZT0-2 of flies with *R20A02-AD;R18H11-DBD dTrpA1* or *iso31 dTrpA1* (Day1) | Unpaired t-test | 0.0756 |  | 64 per genotype |
|  | Sleep bout number at ZT12-24 of flies with *R20A02-AD;R18H11-DBD dTrpA1* or *iso31 dTrpA1* (Day1) | Unpaired t-test | <0.0001 |  | 64 per genotype |
|  | Sleep bout number at ZT0-2 of flies with *R20A02-AD;R18H11-DBD dTrpA1* or *iso31 dTrpA1* (Day2) | Unpaired t-test | 0.3359 |  | 64 per genotype |
|  | Sleep bout number at ZT12-24 of flies with *R20A02-AD;R18H11-DBD dTrpA1* or *iso31 dTrpA1* (Day2) | Unpaired t-test | 0.6643 |  | 64 per genotype |
| S2f | Active time at ZT0-12 of flies with *R20A02-AD;R18H11-DBD dTrpA1* or *iso31 dTrpA1* | Unpaired t-test | 0.0013 |  | 64 per genotype |
|  | Active time at ZT12-24 of flies with *R20A02-AD;R18H11-DBD dTrpA1* or *iso31 dTrpA1* | Unpaired t-test | 0.0046 |  | 64 per genotype |
| S2g | Activity/min awake of flies with *R20A02-AD;R18H11-DBD dTrpA1* or *iso31 dTrpA1* | Unpaired t-test | 0.0005 |  | 64 per genotype |
